# Assessment of foot and ankle muscle strength using hand held dynamometry in patients with established rheumatoid arthritis

**DOI:** 10.1186/1757-1146-6-S1-O4

**Published:** 2013-05-31

**Authors:** Matthew Carroll, Angela Brenton-Rule, William Joyce, Nicola Dalbeth, Keith Rome

**Affiliations:** 1 Health & Rehabilitation Research Institute, AUT University, Auckland, 0627, New Zealand; 2 School of Medicine, University of Auckland, Auckland, New Zealand

## Background

The foot and ankle are frequently affected in patients with rheumatoid arthritis (RA). One of the negative consequences of RA on the physical function of patients is a decrease in muscle strength. However, little is known about foot and muscle strength in this population. The aim of the study was to evaluate significant differences in foot and ankle muscle strength between patients with established RA against age and sex-matched controls using hand-held dynamometry.

## Methods

The maximal muscle strength of ankle plantarflexion, dorsiflexion, eversion and inversion was assessed in 14 patients with RA, mean (SD) disease duration of 22 (14.1) years, and 20 age and sex-matched control participants using hand-held dynamometry.

## Results

Significant differences were observed in muscle strength between the two groups in plantarflexion (p = 0.00), eversion (p = 0.04) and inversion (p = 0.01). No significant differences was found in dorsiflexion (p>0.05). The patients with RA displayed a significantly lower plantarflexion-dorsiflexion ratio than the control participants (p = 0.03).

## Conclusion

The results from this study showed that the RA patients displayed a significant decrease in ankle dorsiflexion, eversion and inversion when compared to the non-RA control group suggesting that foot and ankle muscle strength may be affected by the pathological processes in RA. Monitoring muscle strength in RA patients provides the clinician insight into disease progression within the RA patient.

